# Global Loss of *Bmal1* Expression Alters Adipose Tissue Hormones, Gene Expression and Glucose Metabolism

**DOI:** 10.1371/journal.pone.0065255

**Published:** 2013-06-04

**Authors:** David John Kennaway, Tamara Jayne Varcoe, Athena Voultsios, Michael James Boden

**Affiliations:** Robinson Institute, University of Adelaide, Adelaide, South Australia, Australia; Nihon University School of Medicine, Japan

## Abstract

The close relationship between circadian rhythm disruption and poor metabolic status is becoming increasingly evident, but role of adipokines is poorly understood. Here we investigated adipocyte function and the metabolic status of mice with a global loss of the core clock gene *Bmal1* fed either a normal or a high fat diet (22% by weight). *Bmal1* null mice aged 2 months were killed across 24 hours and plasma adiponectin and leptin, and adipose tissue expression of *Adipoq*, *Lep*, *Retn* and *Nampt* mRNA measured. Glucose, insulin and pyruvate tolerance tests were conducted and the expression of liver glycolytic and gluconeogenic enzyme mRNA determined. *Bmal1* null mice displayed a pattern of increased plasma adiponectin and plasma leptin concentrations on both control and high fat diets. *Bmal1* null male and female mice displayed increased adiposity (1.8 fold and 2.3 fold respectively) on the normal diet, but the high fat diet did not exaggerate these differences. Despite normal glucose and insulin tolerance, *Bmal1* null mice had increased production of glucose from pyruvate, implying increased liver gluconeogenesis. The *Bmal1* null mice had arrhythmic clock gene expression in epigonadal fat and liver, and loss of rhythmic transcription of a range of metabolic genes. Furthermore, the expression of epigonadal fat *Adipoq*, *Retn*, *Nampt*, *AdipoR1* and *AdipoR2* and liver *Pfkfb3* mRNA were down-regulated. These results show for the first time that global loss of *Bmal1*, and the consequent arrhythmicity, results in compensatory changes in adipokines involved in the cellular control of glucose metabolism.

## Introduction

The links between circadian rhythms of gene expression, hormone secretion and metabolism have emerged over recent years. For example glucose metabolism changes across the day and night in humans [1], [2], while studies in animals have shown that ablation of the suprachiasmatic nucleus (SCN), the brain region responsible for the entrainment of physiological systems to the light/dark cycle, results in the loss of the rhythms of glucose tolerance [3]. *Bmal1* (also known as *Mop3* or *Arntl1)* [4] and *Clock* [5] are recognised as critical components of cellular circadian rhythm generation. The BMAL1/CLOCK heterodimeric protein induces other clock genes (e.g., *Per1, Per2*, *Cry1, Cry2, Nr1d1)* upon binding to E-box elements in the promoters of the genes. After complexing with Casein kinase 1ε/δ, the PER/CRY/Casein kinase complex returns to the nucleus and inhibits the action of BMAL1/CLOCK, thus reducing the expression of the period and cryptochrome genes. NR1D1 plays an important role in the generation of rhythmicity through its repression of *Bmal1* expression. We [6], [7] and others [8]–[11] have shown that disruption of endogenous cellular rhythmicity via mutation of *Clock* is associated with alterations in metabolism. The *Clock* ^Δ*19*^ mutants produce a protein that can bind to BMAL1, but not induce gene expression, resulting in a loss of rhythmic gene expression in peripheral tissues, but not the SCN [12]. This mutation has been shown to be associated with obesity [10], hyperinsulinaemia [10], decreased glucose tolerance [7], [11], increased insulin sensitivity [7], [11], hyper-lipidaemia, and decreased plasma free fatty acids [7]. The mutant phenotype does, however, depend upon the background strain of the mice studied. It is known that some of the functions of *Clock* in certain tissues (e.g., brain) are rescued in *Clock* ^Δ*19*^ mutant mice and *Clock* null mice by a homologue, *Npas2* [13]–[15]. The metabolic consequences of global rhythm disruption have been studied by several laboratories, but there are still large gaps in our knowledge. The best candidate for investigating global arrhythmia is the *Bmal1* null mouse, originally produced by the Bradfield laboratory [4], with another line reported recently in Japan [16]. Unlike *Clock* ^Δ*19*^ mutant or *Clock* null mice, *Bmal1* null mice have poorly entrained wheel running behaviour under a normal photoperiod and are arrhythmic in continuous darkness [4]. Moreover, there is no evidence that *Bmal2* rescues the functions of *Bmal1* in the *Bmal1* null mouse [4], [17]. Interestingly *Bmal1* null mice are infertile [18], [19] and as they age, develop a range of physiological deficits including arthropathy [20] and altered cardiovascular function [21].

In this study we report for the first time the effects of loss of *Bmal1* expression on plasma adipokine levels (adiponectin and leptin) and adipose tissue expression of *adiponectin* (*Adipoq*), *leptin* (*Lep*), *resistin* (*Retn*) and *visfatin* (*Nampt*) mRNA in young animals (2 months of age), before any confounding pathology was likely to emerge. In addition we report the effects of global loss of *Bmal1* expression on glucose, insulin and pyruvate tolerance tests and expression of liver glycolytic and gluconeogenic enzyme mRNA. Finally we investigated the metabolic impact of a high fat diet on body and adipose tissue weight, plasma metabolites, insulin and adipokines in *Bmal1* null mice.

## Methods

The founder *Bmal1* null mice [4] were generously provided by Dr C Bradfield (University of Wisconsin Medical School, Madison, WI, USA) and were subsequently maintained as a heterozygous line on the original mixed background (C57Bl/6 and 129/SV). The study was approved by the Animal Ethics Committee of the University of Adelaide. Animals were maintained on a 12 h light:12 h darkness photoperiod (lights off at 2000 h) in the University of Adelaide Medical School Specific Pathogen Free Animal House, and were provided with the control diet of standard mouse chow (7% fat (wt/wt), Ridley AgriProducts, Melbourne, Australia) and water *ad libitum*. The genotypes of the offspring were determined as previously described by PCR of tail DNA [4].

Groups of *Bmal1* null male mice and their wild-type litter mate controls (4 mice per time point) were killed by decapitation at 2 months of age every four hours across 24 hours at 0800 h, 1200 h, 1600 h, 2000 h, 2400 h and 0400 h. Blood was collected into heparinised tubes and plasma harvested for metabolite and hormone assays. Liver and epigonadal fat were rapidly dissected and immediately placed in RNAlater® (Ambion, Austin, TX) and then stored at −20°C until processing. An additional group of 6 month old male *Bmal1* null and wild-type mice (4–9 mice per time point) were killed at the same times of day and blood collected.

To determine the effects of a high-fat diet on plasma hormones and metabolites, male and female *Bmal1* null and wild-type mice were fed a high-fat diet (22% fat (wt/wt), 0.15% cholesterol, 4.6 kcal/g, SF00-219, Specialty Feeds, Glen Forrest, Western Australia) or the control diet from 3 to 8 weeks of age (*n = *10–19 mice of each sex per genotype). Animals were weighed and killed during the mid light period (1400 h), trunk blood collected and the epigonadal/retroperitoneal fat pads, testes/uteri and kidneys were dissected and weighed.

### Intraperitoneal Glucose Tolerance Test

Wild-type and *Bmal1* null male mice aged 2 and 6 months (n = 5–6) were maintained on the control diet, fasted overnight and injected with glucose (1 mg/g body weight; i.p.; Sigma Chemical, St Louis, MO) starting 2 h after the lights were turned on as previously described [7]. Blood was obtained from the tail vein before and 30 and 60 minutes after glucose administration for the determination of blood glucose.

### Intraperitoneal Pyruvate Tolerance Test

Wild-type and *Bmal1* null 2 month old male mice (n = 6) and female mice (n = 5–7) were maintained on the control diet, fasted overnight and injected with sodium pyruvate (2 mg/g body weight; i.p.; Sigma Chemical, St Louis, MO) starting 2 h after the lights were turned on as previously described [22]. Blood was obtained from the tail vein before and 15, 30, 60, 90, 120 minutes after pyruvate administration for the determination of blood glucose.

### Intraperitoneal Insulin Tolerance Test

Wild-type and *Bmal1* null male and female mice (n = 6 per gender) aged 2 months were maintained on the control diet and had food withheld for 2 hours before injection of insulin (0.75 IU/kg body weight; Actrapid; Novo Nordisk Pharmaceuticals Pty. Ltd., Baulkham Hills, Australia) 2–3 h after lights on as previously described [7]. Blood was obtained from the tail vein before and 30, 60, 90 and 120 minutes after insulin administration for the determination of blood glucose.

### Real-time RT-PCR

To investigate the expression of clock and clock controlled genes in the liver and adipose tissue total mRNA was extracted, reverse transcribed and amplified by real time PCR. Some of the primers have been used previously by our group [6], [13], but for completeness all primers used have been listed in Table 1. The calibrator sample was designated as the most highly expressed time point for each gene of interest in the wild-type mice and given a relative expression of 1.

**Table 1:** Primers used.

Gene	Genesymbol	AccessionNumber		Primers	Amplicon Length
*β-actin*	*β-actin*	NM031144	F	CCTCTGAACCCTAAGGCCAA	90 bp
			R	AGCCTGGATGGCTACGTACA	
*Adiponectin*	*Adipoq*	NM_009605	F	TGTTGGAATGACAGGAGCTGAA	104 bp
			R	CACTGAACGCTGAGCGATACA	
***Adiponectin receptor 1***	***AdipoR1***	**NM_028320**	F	GGAGGGACGTTGGAGAGTCAT	105 bp
			R	GCCCGAAAGGAGGGCATA	
***Adiponectin receptor 2***	***AdipoR2***	NM_197985	F	GCTCCTACAGGCCCATCATG	103 bp
			R	CCAATCCGGTAGCACATCGT	
***Bmal1***	***Bmal1***	**AB015203**	F	GTCGAATGATTGCCGAGGAA	101 bp
			R	GGGAGGCGTACTTGTGATGTTC	
***Fructose-1,6-bisphosphatase 1***	***Fbp1***	**NM_019395**	F	CCCGTCCATTGGAGAATTCAT	101 bp
			R	GGTCAAAGTCCTTGGCATAACC	
***Glucokinase***	*Gck*	NM_010292	F	TTTGTGTCGCAGGTGGAGAG	102 bp
			R	CACAATGTCGCAGTCGGC	
***Glucose-6-phosphatase***	***G6pc***	**NM_008061**	F	CTTAAAGAGACTGTGGGCATCAA	101 bp
			R	AATACGGGCGTTGTCCAAAC	
*Leptin*	*Lep*	NM_008493	F	CAGCCTGCCTTCCCAAAA	137 bp
			R	CATCCAGGCTCTCTGGCTTCT	
***Nuclear receptor subfamily 1, group D, member 1 (Rev erb α)***	***Nr1d1***	**NM_145434**	F	TCCAGTACAAACGGTGTCTGAA	101 bp
			R	GCCAACGGAGAGACACTTCTTG	
***Period2***	***Per2***	**NM_011066**	F	AGGCACCTCCAACATGCAA	140 bp
			R	GGATGCCCCGCTTCTAGAC	
*Peroxisome proliferator-activated receptor gamma*	*Pparγ*	NM_011146	F	CGCTGATGCACTGCCTATGA	101 bp
			R	AGAGGTCCACAGAGCTGATTCC	
***6-phosphofructo-2-kinase/fructose-2,6-biphosphatase 3***	***Pfkfb3***	NM_133232	F	GCAAGAAGTTCGCCAATGC	103 bp
			R	TCCGCGGTCTGAATGGTACT	
***Phosphoenolpyruvate carboxykinase 1***	***Pck1***	NM_011044	F	GTTCCCAGGGTGCATGAAAG	107 bp
			R	AGGGCGAGTCTGTCAGTTCAA	
*Resistin*	*Retn*	NM_022984	F	CCTTTTCTTCCTTGTCCCTGAA	101 bp
			R	ACAGGGAGTTGAAGTCTTGTTTGAT	
*Visfatin*	*Nampt*	NM_021524	F	TTTTGAACACATAGTAACACAGTTCTCATC	101 bp
			R	GGTCTTCACCCCATATTTTCTCA	

### Hormone and Metabolite Assays

Plasma glucose and free fatty acids were measured enzymatically [7]. Plasma triglycerides were measured with a Hitachi 912 automated sample system using a kit as per the manufacturer’s instructions (Roche Diagnostics, Australia). Plasma insulin, adiponectin and leptin were assayed by radioimmunoassay [7].

### Statistics

Hormone, body weight, organ weight and gene expression data were analyzed by univariate ANOVA (SPSS v17), using genotype and time of day as the dependent variables followed by *post hoc* analysis using the Bonferroni correction for multiple comparisons. For the body composition, hormone and gene expression data collected across 24 h, the Estimated Marginal Means are reported in the text for the various comparisons. To determine whether hormone and gene expression data were rhythmic, i.e., fitted a sine curve, the data were analyzed using CircWaveBatch; http://hutlab.nl/ [23].

## Results

### Plasma Glucose, Free Fatty Acids and Hormones

**Figure 1:**
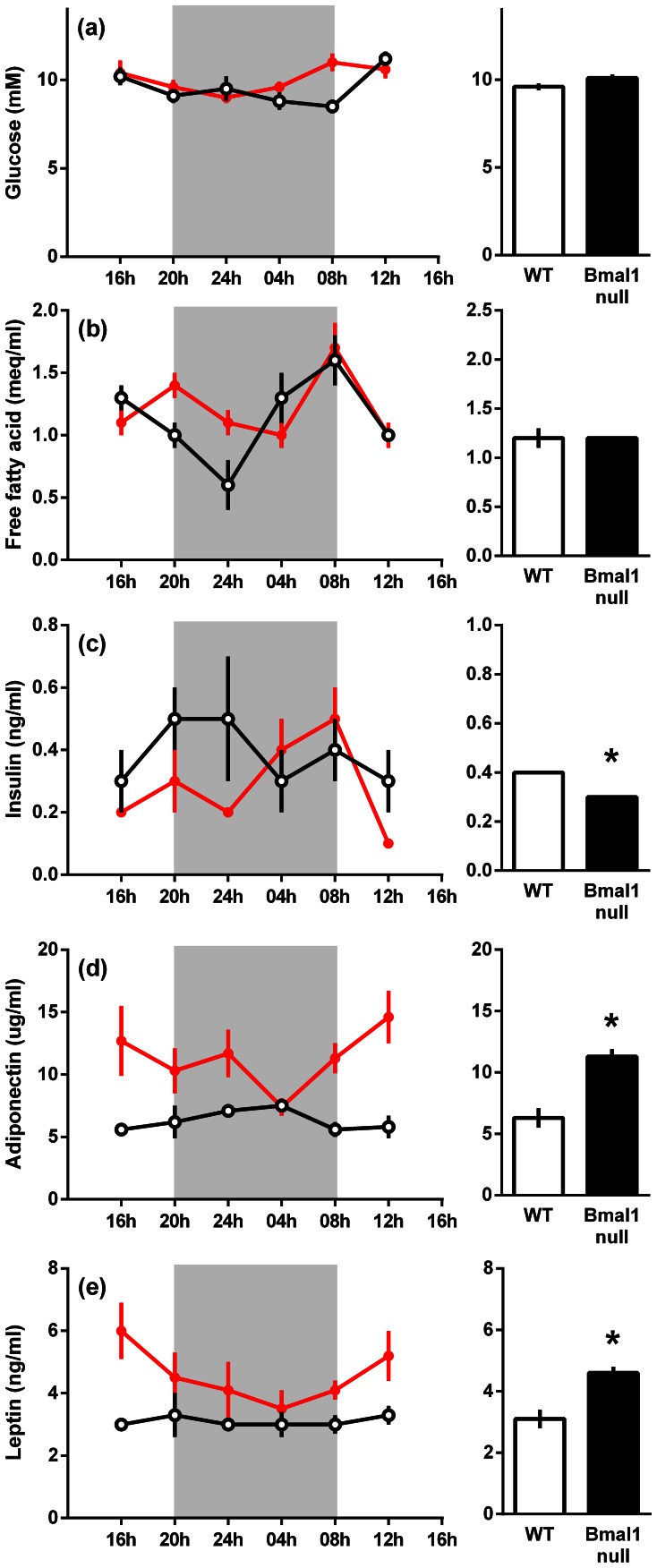
Plasma metabolites, insulin, and adipokines in 8 week old wild-type and *Bmal1* null mice. Plasma glucose (a), free fatty acids (b), insulin (c), adiponectin (d) and leptin (e) levels. Data are the mean ± s.e.m. for *n = *4 mice of each genotype at each time point, wild-type mice (open circles) and *Bmal1* null mice (closed circles). The accompanying histograms represent the estimated marginal means ± s.e.m. of the individual gene expression as calculated from the ANOVA. The shaded areas represent the period of darkness. The symbol * indicates that there was a significant difference (*P*<0.05) between the genotypes.

Plasma glucose and free fatty acids were not different between wild-type and *Bmal1* null mice, however, plasma insulin was lower (P<0.02) and adiponectin (P<0.001) and leptin (P<0.001) were higher in *Bmal1* null compared to wild-type mice (Fig. 1).

Similar differences in plasma insulin and adipokines between *Bmal1* null mice and wild-type mice were observed in 6 month old mice. In particular the *Bmal1* null mice had lower plasma insulin (1.02±0.12 ng/ml in wild-type mice vs. 0.31±0.12 ng/ml in *Bmal1* null mice), higher adiponectin (5.9±0.8 µg/ml in wild-type mice vs.15.7±0.8 µg/ml in *Bmal1* null mice) and higher leptin (0.8±0.1 ng/ml in wild-type mice vs. 2.8±0.2 ng/ml in *Bmal1* null mice) than wild-type mice.

In summary, across 24 hours, plasma insulin was lower and adiponectin and leptin were both higher in 2 month old male *Bmal1* null mice compared to wild-type mice while plasma glucose and free fatty acids were unchanged. These differences persisted in 6 month old mice.

### Intraperitoneal Glucose Tolerance Tests

At 2 and 6 months of age, male *Bmal1* null and wild-type mice had similar fasting blood glucose (data not shown). The peak glucose levels and area under the curve during the glucose tolerance tests in wild-type and *Bmal1* null mice at 2 and 6 months of age were not different (data not shown).

### Intraperitoneal Insulin Tolerance Tests

At 2 months of age, male *Bmal1* null mice had a trend to decreased blood glucose (P = 0.06; Fig. S1) prior to the insulin injection, but the subsequent decrease was similar for the wild-type and *Bmal1* null mice. Furthermore, both groups had a similar counter-regulatory rebound of blood glucose. Similarly there was no difference in the glucose response to insulin in female wild-type and *Bmal1* null mice.

### Intraperitoneal Pyruvate Tolerance Tests

**Figure 2:**
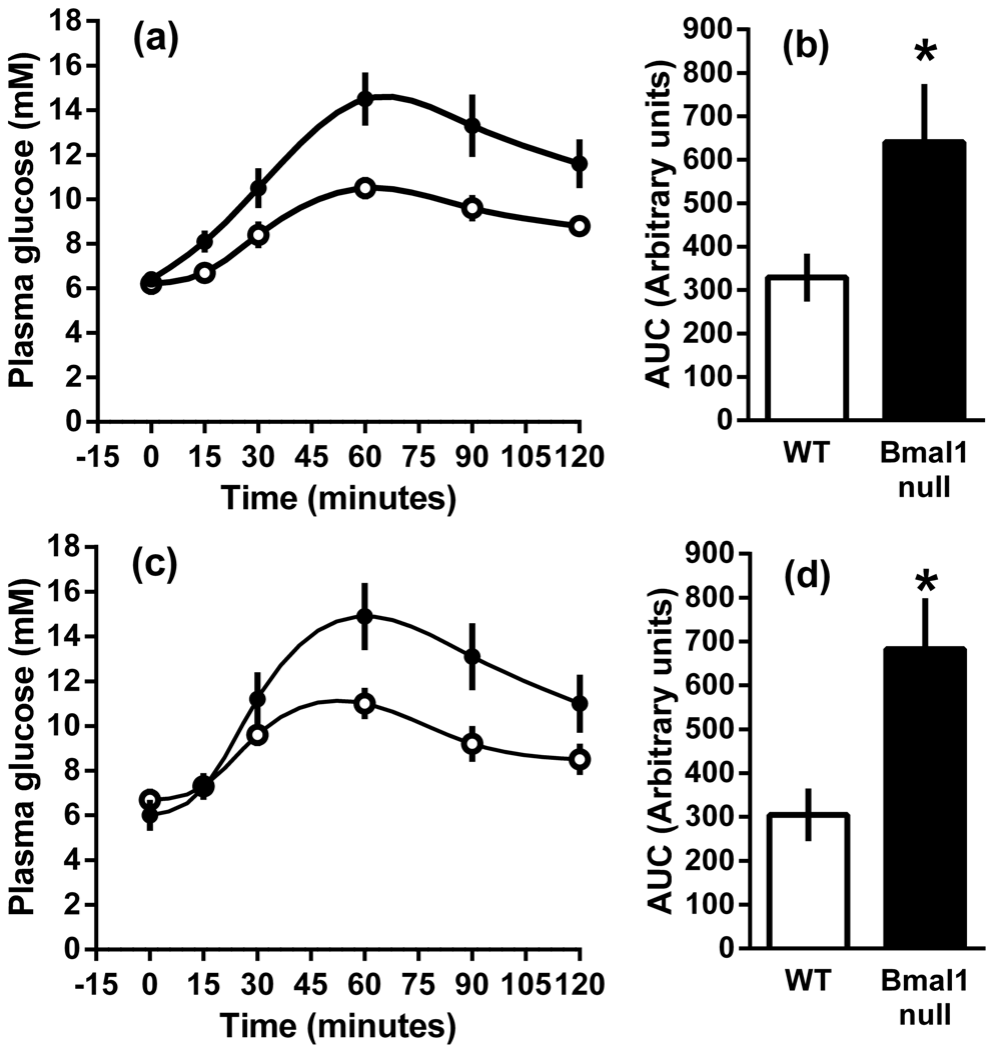
The blood glucose response to the intra peritoneal administration of pyruvate (2 mg/g) to male and female wild-type and *Bmal1* null mice fed a normal rodent diet. The mean ± s.e.m. plasma glucose levels are shown for male (a) and female (c) wild-type (open circles) and *Bmal1* null (closed circles) mice. The mean area under the curve (± SEM) of the plasma glucose profiles up to 120 min post injection (b, d).

At 2 months of age, administration of pyruvate resulted in increased blood glucose levels in both male and female *Bmal1* null mice compared to wild type mice (Fig. 2). The blood glucose levels failed to return to the baseline within 120 minutes in either the wild-type or *Bmal1* null mice, but was significantly higher at 120 minutes post injection in *Bmal1* null mice compared to wild-type mice males (P<0.05), but not females.

### Effect of a High Fat Diet on Body Weight and Fat Depots

**Figure 3:**
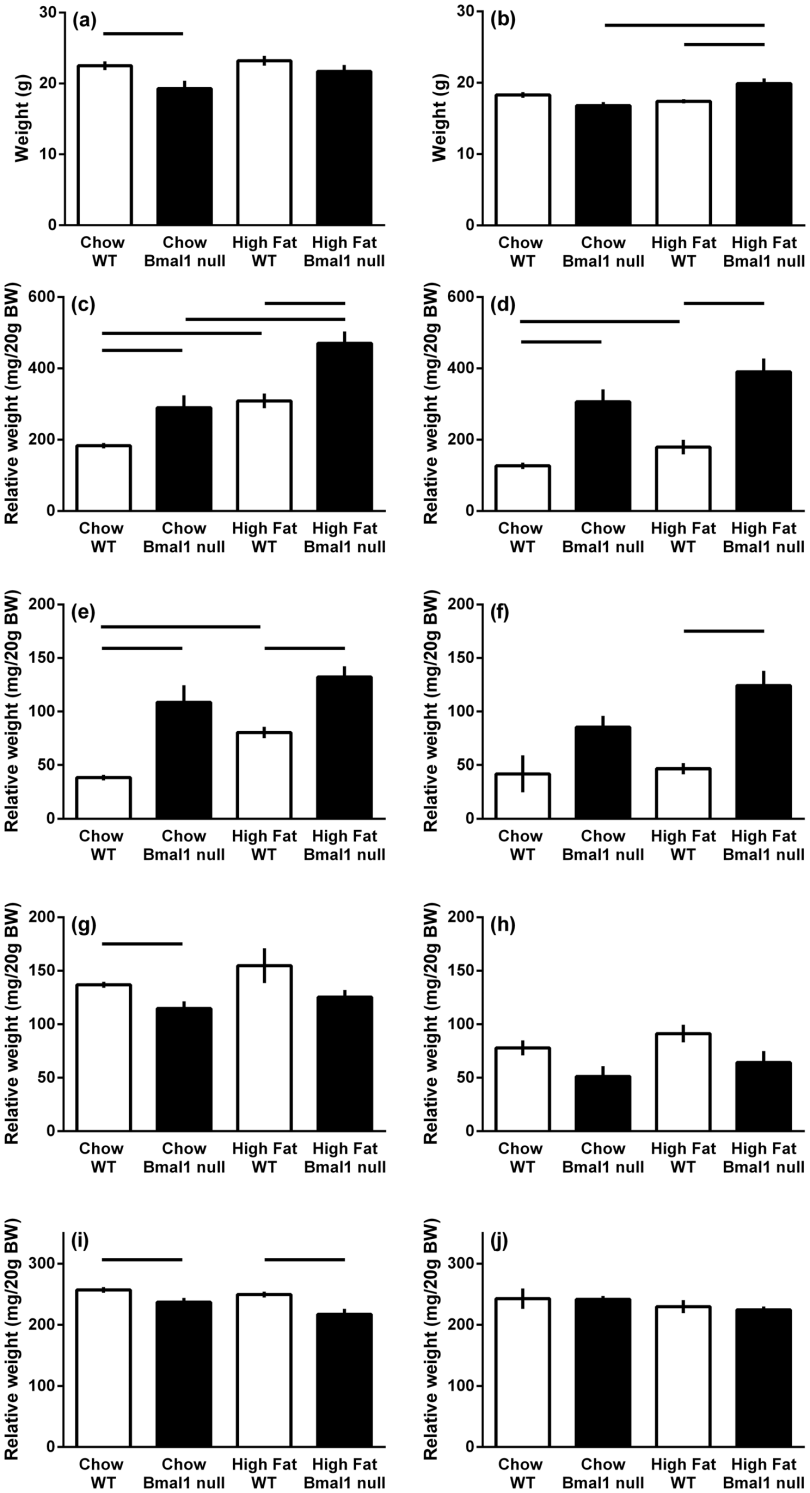
The body composition of male and female wild-type and *Bmal1* null mice fed either normal rodent chow or a high fat diet (22% fat). The data are the means (grams or grams per 20 grams body weight ± s.e.m.; *n = *13–19 mice per group). Body weight (a, b), epigonadal fat pad (c, d), retroperitoneal fat pad (e, f), testis/uterus (g, h) and kidney (i, j). Males (a, c, e, g, i); females (b, d, f, h, j). Bars above the histogram define the difference between the groups with significant difference set to P<0.0125.

Following five weeks on the control diet, body weight of the male *Bmal1* null mice were 14% lower than wild-type mice (P<0.005). On the high fat diet, male *Bmal1* null mice gained weight such that they were no longer lighter than wild type mice (Fig. 3). Wild type mice had increased epididymal and retroperitoneal fat pads when placed on a high fat diet (P<0.001), while in the *Bmal1* null mice only the epididymal fat pads were increased for animals on the high fat diet (P<0.001). Male *Bmal1* null mice had 58% and 52% more epigonadal fat and 182% and 64% more retroperitoneal fat per gram of body weight than wild-type mice maintained on the normal chow and high fat diet respectively (P<0.001). When the epigonadal and retroperitoneal fad pad weights were combined, male *Bmal1* null mice had 1.8 fold more fat than the wild-type mice on the chow diet and 1.5 fold more when on the high fat diet. Testes weight in male *Bmal1* null mice were reduced compared to the wild type mice only when maintained on the chow diet (P<0.001) and kidney weight in the *Bmal1* null males was reduced compared to wild type on both diets (P<0.01).

Body weights of female *Bmal1* null mice on a high fat diet were increased when compared to chow fed *Bmal1* females and wild type females placed on a high fat diet (Fig. 3, P<0.01). Female *Bmal1* null mice had 141% and 117% more epigonadal fat per gram of body weight than wild-type mice maintained on the normal chow and high fat diet respectively (P<0.001). *Bmal1* null mice on a high fat diet had more retroperitoneal fat compared to high fat diet wild type mice (P<0.001) and there was a trend for increased adiposity compared to wild type for *Bmal1* null mice on a chow diet and when comparing *Bmal1* null females on a high fat vs chow diet (P = 0.016 and P = 0.014 respectively). When the epigonadal and retroperitoneal fad pad weights were combined, female *Bmal1* null mice had 2.3 fold more fat than the wild-type mice on the chow diet and 2.3 fold more when on the high fat diet. While showing a trend to smaller uteri in chow fed animals (P = 0.018), female *Bmal1* null mice uteri were not lighter than wild-type mice maintained on the normal chow or high fat diet (P>0.05), nor were kidney weights effected.

In summary, male *Bmal1* null mice weighed less than wild-type mice when maintained on the chow diet but caught up when placed on the high fat diet, whereas female *Bmal1* null mice had comparable body weight on a normal chow diet, but weighed more when maintained on a high fat diet compared to chow fed *Bmal1* null mice or high fat fed wild type mice. For both control and high fat diets, *Bmal1* null male and female mice had a greater degree of adiposity than wild type mice.

### Effect of a High Fat Diet on Plasma Metabolites and Hormones at Mid-light

**Figure 4:**
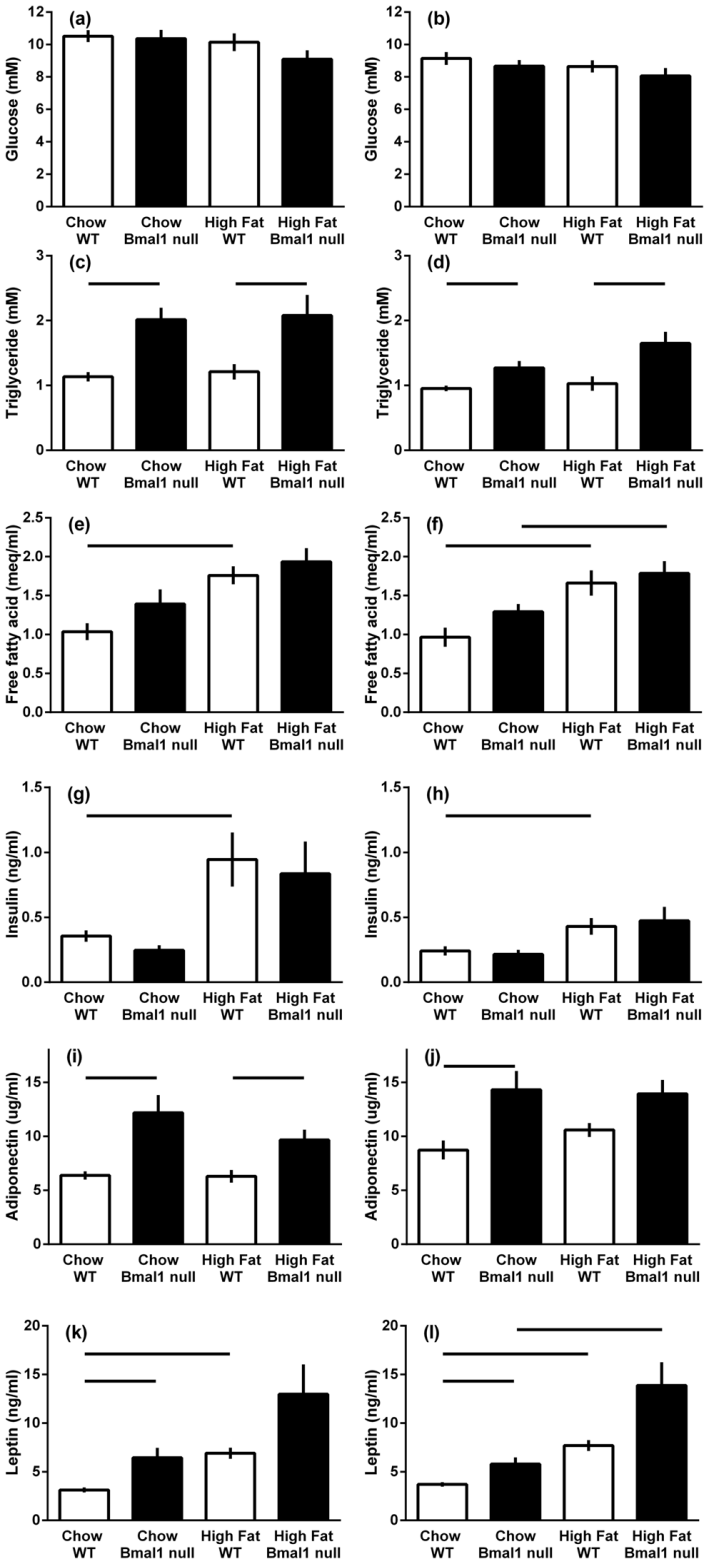
The plasma metabolites and adipokines of male and female wild-type and *Bmal1* null mice fed either normal rodent chow or a high fat diet. The data are the means ± s.e.m. (*n = *9–19 mice per group). Plasma glucose (a, b), triglycerides (c, d), free fatty acids (e, f), insulin (g, h), adiponectin (i, j) and leptin (k, l) levels. Males (a, c, e, g, i); females (b, d, f, h, j). Bars above the histogram define the difference between the groups with significant difference set to P<0.0125.

When the male mice were killed at mid-light (1400 h), there was no difference in plasma glucose, free fatty acids and insulin between wild-type and *Bmal1* null mice, however, plasma triglyceride (78% and 72%) and adiponectin (91% and 51%) were increased in *Bmal1* null mice on chow diet and high fat diet respectively (Fig. 4; P<0.01). The high fat diet did not affect plasma glucose, triglyceride or adiponectin, but increased circulating NEFA (by 70%) and insulin (by 165%) in the wild type mice. Plasma leptin was increased in *Bmal1* compared to wild types on chow diet, and between chow and high fat fed wild type mice (P<0.001).

When the female mice were killed at mid-light (1400 h), there was no difference in plasma glucose, NEFA and insulin between wild-type and *Bmal1* null mice, however adiponectin (by 63%) and leptin (by 56%) were increased in *Bmal1* null mice on chow diet, and plasma triglyceride was increased in *Bmal1* null mice on both chow (33%) and high fat diets (by 33% and 60% respectively, P<0.01). The high fat diet did not affect plasma glucose, triglyceride or adiponectin, but increased NEFA in the wild type (72%) and *Bmal1* null mice (38%), increased circulating insulin by 77% in wild type mice and increased circulating leptin in both wild type and *Bmal1* null mice (139% and 108% respectively, P<0.01).

### Epigonadal Fat Gene Expression

**Figure 5:**
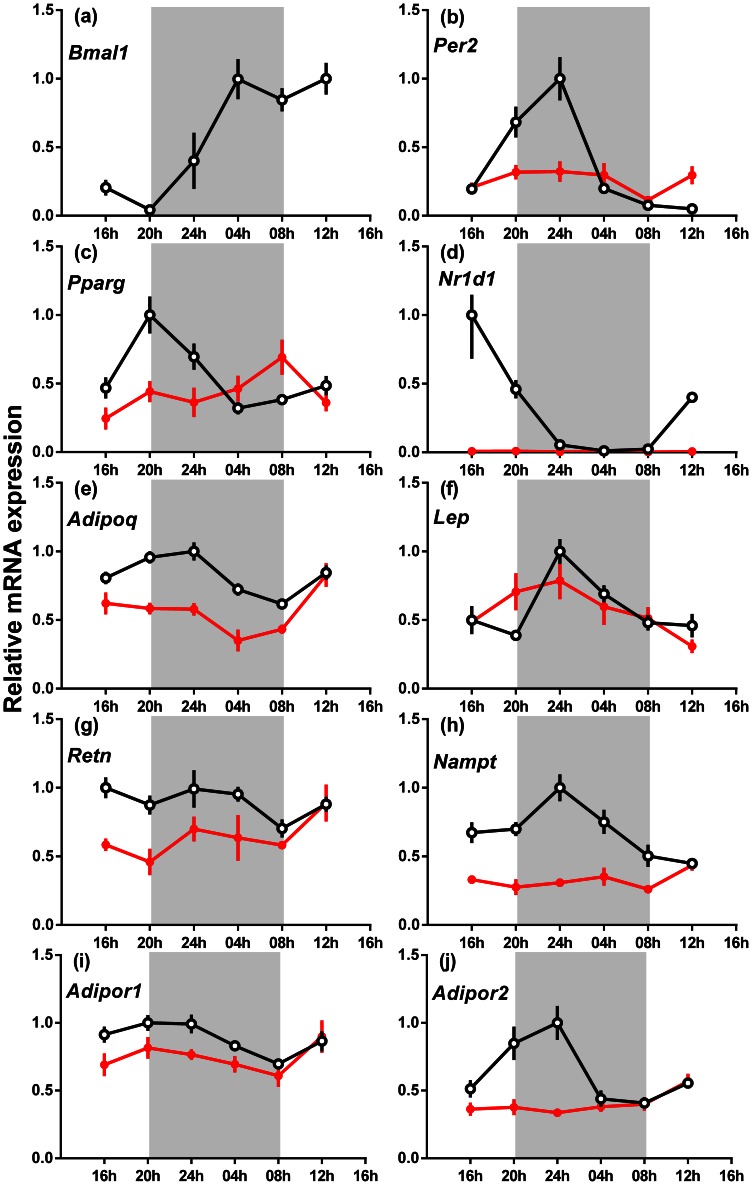
The relative gene expression across 24 h of clock and other genes in the epigonadal adipose tissue of male wild-type and *Bmal1* null mice fed a normal rodent diet. (a) *Bmal1*, (b) *Per2*, (c) *Pparγ*, (d) *Nr1d1*, (e) *Adipoq*, (f) *Lep* (g) *Retn,* (h) *Nampt* (i) *Adipor1* and (j) *Adipor2.* The data are the relative expression for each gene compared to *Actin* mRNA (mean ± s.e.m., n = 4 for each genotype), wild-type mice (open circles) and *Bmal1* null mice (closed circles). The highest expression of each gene for wild-type mice was set at one. The apparent absence of an SEM bar indicates that it is obscured by the symbol. The shaded areas represent the period of darkness.

In the epigonadal fat, overall expression of *Per2* (−30%), *Pparγ* (24%), *Nr1d1* (−98%), *Adipoq* (−31%), *Retn* (−29%), *Nampt* (−52%), *Adipor1* (−16%) and *Adipor2* (−35%) mRNA was decreased (P<0.05), but *Lep* mRNA was unchanged (P>0.05) in male *Bmal1* null mice compared to the wild-type mice (Fig. 5). Expression of *Bmal1*, *Per2*, *Pparγ, Nr1d1*, *Adipoq*, *Lep*, *Adipor1* and *Adipor2* mRNA was rhythmic in wild-type mice (fitted significantly to a sine curve), but *Retn* and *Nampt* mRNA expression was arrhythmic. Expression of all genes analysed in male *Bmal1* null mice was arrhythmic except for *Lep* mRNA (P<0.05).

### Liver Gene Expression

**Figure 6:**
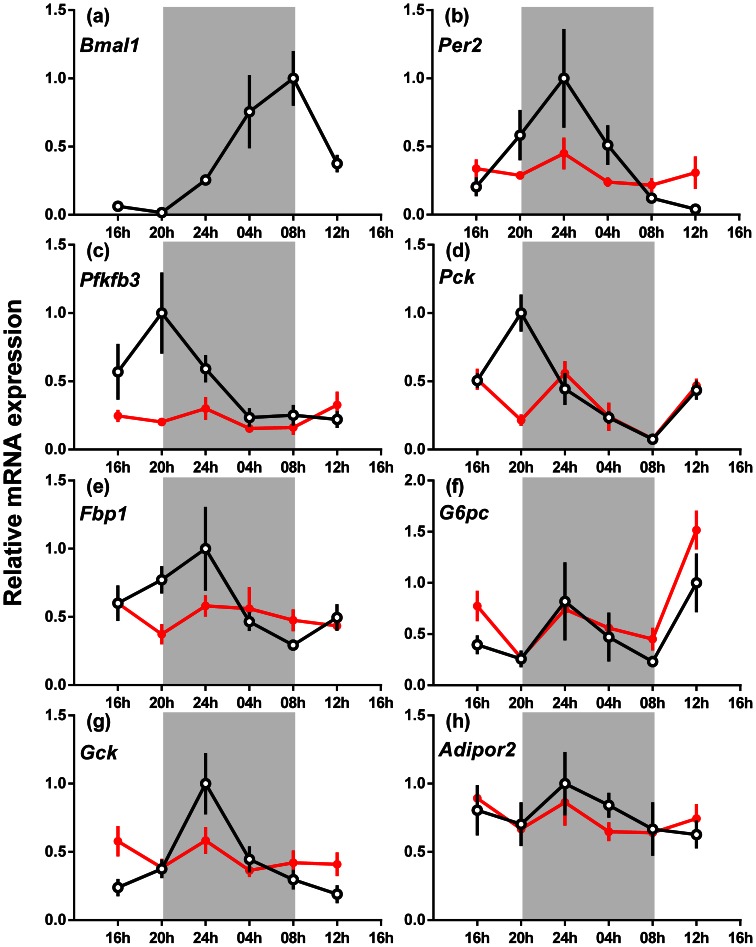
The relative gene expression across 24 h of clock and liver enzyme genes in the liver of male wild-type and *Bmal1* null mice fed a normal rodent diet. (a) *Bmal1*, (b) *Per2*, (c) *Pfkfb3*, (d) *Pck*, (e) *Fbp1*, (f) *G6pc,* (g) *Gck* and (h) *Adipor2.* The data are the relative expression for each gene compared to *Actin* mRNA (mean ± s.e.m., n = 4 for each genotype), wild-type mice (open circles) and *Bmal1* null mice (closed circles). The highest expression of each gene for wild-type mice was set at one. The apparent absence of an SEM bar indicates that it is obscured by the symbol. The shaded areas represent the period of darkness.

In the liver overall Per2, Pck1 (*phosphoenolpyruvate carboxykinase 1*), Fbp1 (fructose-1,6-bisphosphatase 1), G6pc (glucose-6-phosphatase ), Gck (*glucokinase*) and Adipor2 mRNA expression did not vary with genotype, whereas Pfkfb3 (6-phosphofructo-2-kinase/fructose-2,6-biphosphatase 3) mRNA was decreased by 25% in male Bmal1 null mice compared to wild type mice (Fig. 6; P<0.05). Bmal1, Per2, Pfkfb3, Pck1, Fbp1 and Gck mRNA was expressed rhythmically in the wild type mice (P<0.05), whereas G6pc and Adipor2 mRNA expression was arrhythmic. Expression of all genes analysed in male Bmal1 null mice was arrhythmic.

The gene expression levels for the genes involved in the glycolysis and gluconeogenesis pathways (*Gck*, *Pck1*, *Pfkfb3*, *Fbp1* and *G6pc*) were also analysed (ANOVA) using only the 0800 h and 1200 h time points (light period), the time around which the glucose and insulin tolerance tests were performed. In contrast to the 24 hour analysis, expression of *Gck* and *G6pc* were increased 1.7 fold (P = 0.05) and 1.6 fold (P = 0.09) respectively in *Bmal1* null mice at these times.

In summary, across 24 hours, *Pfkfb3* mRNA was decreased, but expression of *Per2*, *Gck*, *Pck1*, *Fbp1*, *G6pc* and *Adipor2* mRNA was unchanged. Expression of *Gck* and *G6pc* mRNA was increased during the early light period. The rhythm of expression of *Per2*, *Pfkfb3*, *Pck1*, *Fbp1* and *Gck* mRNA evident in wild-type mice was absent in male *Bmal1* null mice.

## Discussion

In this study we show for the first time that global disruption of gene rhythmicity in both male and female *Bmal1* null mice alters plasma levels of adiponectin and leptin and adipokine gene expression (*Adipoq*, *Nampt* and *Retn* mRNA). At 2 and 6 months of age, plasma adiponectin and leptin were higher in *Bmal1* null mice than wild-type mice. Although tending to weigh less than the wild-type mice, *Bmal1* null mice had a greater degree of adiposity (1.8 fold and 2.3 fold more fat in males and females respectively). *Adipoq*, *Nampt* and *Retn* mRNA expression was *reduced* in *Bmal1* null compared to the wild-type mice, but their increased adiposity is consistent with the increased secretion of these adipokines. We were not able to measure plasma resistin or visfatin in this study due to the limited volume of plasma available for assay.

The “Western diet” (containing 22% fat and 0.15% cholesterol) used in this study was chosen as we wished to challenge the homeostasis of the *Bmal1* null mice and observe initial stages of loss of metabolic control, which may have been obscured with a more extreme and less physiological diet. When the mice were fed the diet for 5 weeks, the differences in adiposity between the *Bmal1* null and wild-type mice was maintained but not increased, suggesting the *Bmal1* null mice are not more sensitive to this metabolic challenge. There was a pattern towards a small increase in body weight in *Bmal1* null mice in response to 5 weeks on the high fat diet, with minimal change in the wild type mice. This was not unexpected given the moderate challenge of the diet, however both *Bmal1* null and wild type mice increased their adiposity by approximately 50%. Both lines showed signs of increasing their plasma free fatty acids and leptin to a similar extent on the diet. In previous studies, the body weight of comparable age *Bmal1* null mice has been reported as normal [24], [25], increased [26] or decreased [16], [27], [28]. Similarly the increased adiposity observed here has been reported previously in some studies, [24], [26], [28], but not by others [4], [16], [29].

In this study, plasma insulin (calculated across 24 hours) was decreased in male *Bmal1* null mice at 2 and 6 months of age, which is consistent with the decreased glucose stimulated insulin section reported by 3 independent groups [11], [16], [25]. Accompanying the secretory defect, glucose intolerance was reported in 2 studies [11], [25] while in another study there was decreased plasma insulin and increased glucose tolerance [24]. We observed neither glucose intolerance nor altered whole body insulin sensitivity in *Bmal1* null mice. A possible explanation for the normal glucose despite decreased insulin secretion in *Bmal1* null mice may relate to the elevated plasma adiponectin which is known to be an insulin sensitising adipokine [30]. In previous studies in our laboratory we found that *Clock* ^Δ*19*^+MEL mutant mice [12] had low/normal plasma glucose and insulin, but were glucose intolerant and insulin sensitive [7]. They also had elevated plasma adiponectin levels and *Adipoq* mRNA expression, but unlike the *Bmal1* null mice, did not show increased adiposity [6]. We hypothesise that both the *Clock* ^Δ*19*^+MEL mutant and *Bmal1* null mice have adapted to a pancreatic insulin secretory defect by altering the levels of adipokines that modulate insulin sensitivity (adiponectin), to maintain glucose homeostasis. Interestingly, *Clock* ^Δ*19*^+MEL mutant mice had low plasma free fatty acids [7] which would also be expected to improve insulin sensitivity, but *Bmal1* null mice did not exhibit this phenotype.

*Bmal1* null mice did not express *Per2* mRNA rhythmically in the liver in contrast to the wild-type mice. These results confirm the original observations, that disruption of *Bmal1* interferes with circadian rhythm generation [4]. Expression of several of the genes for enzymes involved in glycolysis and gluconeogenesis (*Pfkfb3*, *Pck*, *Fbp1*, and *Gck*) were rhythmic in wild-type, but not *Bmal1* null mice. When analysed across 24 hours, the overall expression of these genes was not altered, except for *Pfkfb3* mRNA expression, which was reduced. Expression of *Pck* and *Fbp1* mRNA prior to darkness was clearly reduced in the *Bmal1* null mice, perhaps reflecting a change in feeding pattern caused by the loss of rhythmicity.

The observation in the current study that *Bmal1* null mice have increased pyruvate tolerance was unexpected because a previous study reported that the loss of *Bmal1* resulted in *decreased* pyruvate tolerance when the test was conducted 7 hours after expected lights on (ZT7) [9]. No measurements of either the expression of genes involved in gluconeogenesis or enzyme activity were reported and it was not clear what control strain was used. Nevertheless it was concluded that gluconeogenesis was abolished by deletion of *Bmal1*. More recently Shimba *et al.* [16] reported that the peak blood glucose level was significantly higher and the clearance was slower in *Bmal1* null mice, as compared to those in control mice following a pyruvate challenge test, similar to that observed here. However, Shimba *et al.* [16] reported that the expression of *G6pc* and *Fbp* mRNA in the liver was increased in the *Bmal1* null mice and that liver glucose-6 phosphate was significantly higher than that of control mice, whereas we found the overall 24 hour expression of *Pfkfb3* mRNA to be lower in the *Bmal1* null mice, particularly during the 4 hours before lights off. This discrepancy may be explained by the fact that in the Shimba *et al.* study [16] the gene analyses were conducted on animals that had been fasted for 16 hours prior to tissue collection at ZT10 (2 hours before lights off). In the current study, we collected livers at 6 time points over 24 hours from animals fed *ad libitum*. We found a trend (P = 0.09; 2-way ANOVA) for higher *G6pc* mRNA expression in the livers of *Bmal1* null mice at 0800 h and 1200 h, the time at which the pyruvate tolerance tests were conducted. Thus these results may explain the increase in glucose conversion in male and female *Bmal1* null mice over the wild type mice. The failure of the gene expression data to reach significance in the sub analysis of the 0800 h and 1200 h time points probably reflects the lower power. Future studies on the impact of loss of *Bmal1* on gluconeogenesis should carefully control the time of day and feed access that the analyses are conducted and in addition assess possible changes in enzyme activity.

The observation of increased adiposity in *Bmal1* null mice on normal chow confirms 3 recent reports [16], [24], [28]. While this may be considered confirmatory evidence that disrupted circadian rhythmicity results in obesity as previously hypothesised on the basis of a study in *Clock* ^Δ*19*^ (C57Bl/6) mice [10], there are some caveats to be considered. First the *Clock* ^Δ*19*^+MEL mice [12] which are on a CBA background do not have an obese phenotype nor do they become disproportionately fat when fed a high fat diet [6]. Second, the adipokine profile of the *Bmal1* null mice is not typical since both adiponectin and leptin are elevated. In the case of *Clock* ^Δ*19*^+MEL mice, adiponectin is elevated but not leptin [6]. Adiponectin is normally low and leptin high in obese rodent models. There was no change in adiponectin receptor mRNA expression in liver, but both *Adipor1* and *Adipor2* mRNA expression was decreased in epigonadal fat. This suggests that there are important modifications in adipose tissue that develop in the absence of *Bmal1* or disrupted clock function when the defect is present throughout life. Interestingly, the size of the adipocytes in the wild-type and *Bmal1* null mice were not different (data not shown), suggesting that the lack of *Bmal1* potentiated adipogenesis.

In the current study we identified a profound decrease in *Nr1d1* mRNA expression in the epigonadal fat pads in *Bmal1* null mice. It is well established that CLOCK/BMAL1 heterodimers induce *Nr1d1* mRNA through interactions at an E-box on the promoter [31]. Loss of either *Clock* or *Bmal1* will reduce *Nr1d1* mRNA expression and eliminate its rhythmicity. Adipogenesis is characterised by high *Nr1d1* mRNA expression [32], [33] and *Nr1d1* mRNA is induced by PPARγ [34]. In *Bmal1* null mice, loss of rhythmic *Nr1d1* resulted in arrhythmic *Pparγ* expression but levels of *Pparγ* mRNA remained around the circadian nadir levels. The high degree of adiposity in *Bmal1* null mice would therefore suggest that low constitutive expression of *Nr1d1* may be sufficient to allow adipogenesis and that its rhythmicity is not critical or that the lipolytic pathways in *Bmal1* null mice are also significantly downregulated.

The metabolic phenotype of *Bmal1* null mice is different from that found in *Clock* ^Δ*19*^ mutant mice on either C57Bl/6 [10], IRC [8], [35], [36] or CBA [7] backgrounds. The metabolic phenotype of *Clock* ^Δ*19*^ mutants is one of obesity, hyper-insulinaemia, hyper-lipidaemia, glucose intolerance and increased insulin sensitivity [6], [7], [10], [11], although not all metabolic alterations are observed in mutants on all backgrounds. In the current study we used *Bmal1* null mice that were maintained on a mixed background because we did not wish to confound the impact of *Bmal1* loss with other metabolically important mutations present in inbred strains like the C57/Bl6 mice [37]. We expected that the metabolic consequences for global *Bmal1* null mice with a complete lack of central and peripheral rhythmicity would be exacerbated over and above those observed in *Clock* ^Δ*19*^ mutants. *Bmal1* null mice do not, however, show evidence of a major metabolic disorder. This is not to say that *Bmal1* null mice are normal, since they have a reduced life expectancy [29], are prone to develop arthropathies [20] and are infertile [18], [19]. At least while they are maintained on *ad libitum* feeding, *Bmal1* null mice appear able to maintain normo-glycaemia, despite hypertriglyceridaemia, hyperlipidaemia and hyperleptinaemia. Interestingly despite this potential cardiovascular risk, *Bmal1* null mice have recently been shown to be hypotensive and have lower stress induced alterations in blood pressure [21]. As in many other genetic animal models, *Bmal1* null mice appear to have compensated for the long term lack of *Bmal1* and/or rhythmicity by altering a range of physiological systems including adipose tissue growth, adipokine secretion and feeding behaviour to maintain glucose homeostasis.

Our analyses of the metabolic parameters of the *Bmal1* null model do not discriminate between that caused by arrhythmicity and any non-circadian functions of this gene. Nevertheless, there is mounting evidence for a role of circadian rhythms in the regulation of metabolic function, as demonstrated through a variety of knock out and mutant mice models. Furthermore, from our results, it is clear that removal of the *Bmal1* gene leads to cellular arrhythmicity of not only clock genes, but also genes involved in key metabolic processes including adipogenesis and gluconeogenesis. We believe therefore, that it is this circadian arrhythmicity, at both the cellular and whole organism level, that leads to the metabolic perturbations observed here, rather than any non-clock function of *Bmal1*.

In summary, global loss of *Bmal1*, and the circadian arrhythmicity this creates, leads to increased adiposity when maintained on both control and high fat diets. Furthermore, the *Bmal1* null mice displayed increased plasma adiponectin and leptin levels, despite reduced or normal expression of *Adipoq* and *Lep* mRNA respectively. Presumably the 1.8–2.3 fold increase in adiposity, and hence the greater number of fat cells available for secretion of these hormones, plays a role in the elevated concentrations in plasma. *Bmal1* null mice also displayed an elevated response to pyruvate challenge, which when considered with the normal glucose and insulin tolerance, suggest an increased capacity for gluconeogenesis. Nevertheless, *Bmal1* null mice display normal profiles of plasma glucose. Finally the loss of adipocyte and hepatic rhythmic gene expression and down-regulation of key metabolic genes in both tissues emphasises further the regulatory role *Bmal1* is able to play. Together these results demonstrate that disrupted circadian rhythmicity leads to changes in adipocyte function and compensatory changes in adipokines involved in the cellular control of glucose metabolism, and provides further support for the role of circadian rhythms in the regulation of metabolic homeostasis.

## Supporting Information

Figure S1 **Blood glucose response to intraperitoneal administration of insulin.** Insulin (0.75 IU/kg) was injected into wild-type (open circle) and *Bmal1* null (closed circle) mice and blood glucose measured over the subsequent 2 hours. Data are mean ± SEM (n = 6); (a) males, (b) females.
(TIF)

## References

[1] Van Cauter E, Desir D, Decoster C, Fery F, Balasse EO (1989) Nocturnal decrease in glucose tolerance during constant glucose infusion. J Clin Endocrinol Metab 69: 604–611. 2668321 10.1210/jcem-69-3-604

[2] Van Cauter E, Polonsky KS, Scheen AJ (1997) Roles of circadian rhythmicity and sleep in human glucose regulation. Endocrine Reviews 18: 716–738. 9331550 10.1210/edrv.18.5.0317

[3] la Fleur SE, Kalsbeek A, Wortel J, Fekkes ML, Buijs RM (2001) A daily rhythm in glucose tolerance: a role for the suprachiasmatic nucleus. Diabetes 50: 1237–1243. 11375322 10.2337/diabetes.50.6.1237

[4] Bunger MK, Wilsbacher LD, Moran SM, Clendenin C, Radcliffe LA, et al (2000) Mop3 is an essential component of the master circadian pacemaker in mammals. Cell 103: 1009–1017. 11163178 10.1016/s0092-8674(00)00205-1 PMC3779439

[5] Vitaterna MH, King DP, Chang AM, Kornhauser JM, Lowrey PL, et al (1994) Mutagenesis and mapping of a mouse gene, Clock, essential for circadian behavior. Science 264: 719–725. 8171325 10.1126/science.8171325 PMC3839659

[6] Kennaway DJ, Owens JA, Voultsios A, Wight N (2012) Adipokines and adipocyte function in Clock mutant mice that retain melatonin rhythmicity. Obesity 20: 295–305. 21918578 10.1038/oby.2011.276

[7] Kennaway DJ, Owens JA, Voultsios A, Boden MJ, Varcoe TJ (2007) Metabolic homeostasis in mice with disrupted Clock gene expression in peripheral tissues. American Journal of Physiology 293: R1528–R1537. 17686888 10.1152/ajpregu.00018.2007

[8] Oishi K, Atsumi GI, Sugiyama S, Kodomari I, Kasamatsu M, et al (2006) Disrupted fat absorption attenuates obesity induced by a high-fat diet in Clock mutant mice. FEBS Letters 580: 127–130. 16343493 10.1016/j.febslet.2005.11.063

[9] Rudic RD, McNamara P, Curtis AM, Boston RC, Panda S, et al (2004) BMAL1 and CLOCK, two essential components of the circadian clock, are involved in glucose homeostasis. PLoS Biology 2: e377. 15523558 10.1371/journal.pbio.0020377 PMC524471

[10] Turek FW, Joshu C, Kohsaka A, Lin E, Ivanova G, et al (2005) Obesity and metabolic syndrome in circadian *Clock* mutant mice. Science 308: 1043–1045. 15845877 10.1126/science.1108750 PMC3764501

[11] Marcheva B, Ramsey KM, Buhr ED, Kobayashi Y, Su H, et al (2010) Disruption of the clock components CLOCK and BMAL1 leads to hypoinsulinaemia and diabetes. Nature 466: 627–631. 20562852 10.1038/nature09253 PMC2920067

[12] Kennaway DJ, Voultsios A, Varcoe TJ, Moyer RW (2003) Melatonin and activity rhythm responses to light pulses in mice with the Clock mutation. American Journal of Physiology 284: R1231–1240. 12521925 10.1152/ajpregu.00697.2002

[13] Kennaway DJ, Owens JA, Voultsios A, Varcoe TJ (2006) Functional central rhythmicity and light entrainment, but not liver and muscle rhythmicity, are Clock independent. American Journal of Physiology 291: R1172–R1180. 16709646 10.1152/ajpregu.00223.2006

[14] Debruyne JP, Weaver DR, Reppert SM (2007) CLOCK and NPAS2 have overlapping roles in the suprachiasmatic circadian clock. Nature Neuroscience 10: 543–545. 17417633 10.1038/nn1884 PMC2782643

[15] Debruyne JP, Noton E, Lambert CM, Maywood ES, Weaver DR, et al (2006) A clock shock: mouse CLOCK is not required for circadian oscillator function. Neuron 50: 465–477. 16675400 10.1016/j.neuron.2006.03.041

[16] Shimba S, Ogawa T, Hitosugi S, Ichihashi Y, Nakadaira Y, et al (2011) Deficient of a clock gene, Brain and Muscle Arnt-Like Protein-1 (BMAL1), induces dyslipidemia and ectopic fat formation. PLoS ONE 6: e25231. 21966465 10.1371/journal.pone.0025231 PMC3178629

[17] Shi S, Hida A, McGuinness OP, Wasserman DH, Yamazaki S, et al (2010) Circadian clock gene Bmal1 is not essential; functional replacement with its paralog, Bmal2. Current Biology 20: 316–321. 20153195 10.1016/j.cub.2009.12.034 PMC2907674

[18] Boden MJ, Varcoe TJ, Voultsios A, Kennaway DJ (2010) Reproductive biology of female *Bmal1* null mice. Reproduction 139: 1077–1090. 20200203 10.1530/REP-09-0523

[19] Ratajczak CK, Boehle KL, Muglia LJ (2009) Impaired steroidogenesis and implantation failure in Bmal1−/− mice. Endocrinology 150: 1879–1885. 19056819 10.1210/en.2008-1021 PMC5393263

[20] Bunger MK, Walisser JA, Sullivan R, Manley PA, Moran SM, et al (2005) Progressive arthropathy in mice with a targeted disruption of the Mop3/Bmal-1 locus. Genesis 41: 122–132. 15739187 10.1002/gene.20102

[21] Curtis AM, Cheng Y, Kapoor S, Reilly D, Price TS, et al (2007) Circadian variation of blood pressure and the vascular response to asynchronous stress. Proceedings of the National Academy of Sciences 104: 3450–3455. 10.1073/pnas.0611680104 PMC1802007 17360665

[22] Miyake K, Ogawa W, Matsumoto M, Nakamura T, Sakaue H, et al (2002) Hyperinsulinemia, glucose intolerance, and dyslipidemia induced by acute inhibition of phosphoinositide 3-kinase signaling in the liver. J Clin Invest 110: 1483–1491. 12438446 10.1172/JCI15880 PMC151813

[23] Oster H, Damerow S, Hut RA, Eichele G (2006) Transcriptional profiling in the adrenal gland reveals circadian regulation of hormone biosynthesis genes and nucleosome assembly genes. Journal of Biological Rhythms 21: 350–361. 16998155 10.1177/0748730406293053

[24] Lamia KA, Storch KF, Weitz CJ (2008) Physiological significance of a peripheral tissue circadian clock. Proceedings of the National Academy of Sciences 105: 15172–15177. 10.1073/pnas.0806717105 PMC2532700 18779586

[25] Lee J, Kim MS, Li R, Liu VY, Fu L, et al (2011) Loss of Bmal1 leads to uncoupling and impaired glucose-stimulated insulin secretion in beta-cells. Islets 3: 381–388. 22045262 10.4161/isl.3.6.18157 PMC3329519

[26] Guo B, Chatterjee S, Li L, Kim JM, Lee J, et al (2012) The clock gene, brain and muscle Arnt-like 1, regulates adipogenesis via Wnt signaling pathway. FASEB Journal 26: 3453–3463. 22611086 10.1096/fj.12-205781 PMC6137895

[27] McDearmon EL, Patel KN, Ko CH, Walisser JA, Schook AC, et al (2006) Dissecting the functions of the mammalian clock protein BMAL1 by tissue-specific rescue in mice. Science 314: 1304–1308. 17124323 10.1126/science.1132430 PMC3756687

[28] Hemmeryckx B, Himmelreich U, Hoylaerts MF, Lijnen HR (2011) Impact of clock gene Bmal1 deficiency on nutritionally induced obesity in mice. Obesity 19: 659–661. 21030946 10.1038/oby.2010.266

[29] Kondratov RV, Kondratova AA, Gorbacheva VY, Vykhovanets OV, Antoch MP (2006) Early aging and age-related pathologies in mice deficient in BMAL1, the core componentof the circadian clock. Genes and Development 20: 1868–1873. 16847346 10.1101/gad.1432206 PMC1522083

[30] Rabe K, Lehrke M, Parhofer KG, Broedl UC (2008) Adipokines and insulin resistance. Mol Med 14: 741–751. 19009016 10.2119/2008-00058.Rabe PMC2582855

[31] Triqueneaux G, Thenot S, Kakizawa T, Antoch MP, Safi R, et al (2004) The orphan receptor Rev-erbalpha gene is a target of the circadian clock pacemaker. J Mol Endocrinol 33: 585–608. 15591021 10.1677/jme.1.01554 PMC3770723

[32] Chawla A, Lazar MA (1993) Induction of Rev-ErbA alpha, an orphan receptor encoded on the opposite strand of the alpha-thyroid hormone receptor gene, during adipocyte differentiation. Journal of Biological Chemistry 268: 16265–16269. 8344913

[33] Wang J, Lazar MA (2008) Bifunctional role of Rev-erbalpha in adipocyte differentiation. Mol Cell Biol 28: 2213–2220. 18227153 10.1128/MCB.01608-07 PMC2268425

[34] Fontaine C, Dubois G, Duguay Y, Helledie T, Vu-Dac N, et al (2003) The orphan nuclear receptor Rev-Erbalpha is a peroxisome proliferator-activated receptor (PPAR) gamma target gene and promotes PPARgamma-induced adipocyte differentiation. Journal of Biological Chemistry 278: 37672–37680. 12821652 10.1074/jbc.M304664200

[35] Kudo T, Kawashima M, Tamagawa T, Shibata S (2008) Clock mutation facilitates accumulation of cholesterol in the liver of mice fed a cholesterol/cholic acid diet. American Journal of Physiology 294: E120–E130. 17971517 10.1152/ajpendo.00061.2007

[36] Kudo T, Tamagawa T, Kawashima M, Mito N, Shibata S (2007) Attenuating effect of clock mutation on triglyceride contents in the ICR mouse liver under a high-fat diet. J Biol Rhythms 22: 312–323. 17660448 10.1177/0748730407302625

[37] Toye AA, Lippiat JD, Proks P, Shimomura K, Bentley L, et al (2005) A genetic and physiological study of impaired glucose homeostasis control in C57BL/6J mice. Diabetologia 48: 675–686. 15729571 10.1007/s00125-005-1680-z

